# MRI-based parameters to assess the quality and prognosis of closed reduction in toddlers with developmental dysplasia of the hip

**DOI:** 10.3389/fsurg.2025.1643044

**Published:** 2025-08-26

**Authors:** Jinchao Cao, Yu Li, Junzhong Luo, Zhaosu Zheng, Xuan Wang, Yunshan Su, Jiuhui Han

**Affiliations:** Department of Pediatric Orthopedics, The Third Hospital of Hebei Medical University, Shijiazhuang, China

**Keywords:** developmental dysplasia of the hip, MRI, toddlers, closed reduction, spica cast immobilization

## Abstract

**Background:**

Developmental dysplasia of the hip (DDH) in toddlers (9–18 months) presents unique challenges due to incomplete ossification and anatomical complexity. Traditional imaging modalities, including x-ray, CT, arthrography, and ultrasonography, have limitations in assessing reduction quality. This study introduces a novel approach using only two magnetic resonance imaging (MRI) parameters-axial acetabular femoral head distance (aAFD) and coronal acetabular cartilage head index (CAHI)-to evaluate the quality of closed reduction (CR) and identify risk factors for redislocation.

**Methods:**

We retrospectively reviewed 51 patients (58 hips) who underwent CR for DDH from June 2014 to June 2021. Pre- and post-CR pelvic radiographs assessed dysplasia grade, acetabular index (AI), and avascular necrosis (AVN). MRI, performed within three days post-CR, evaluated hip reduction quality using aAFD and CAHI. The reliability of these indices and their association with redislocation risk were analyzed.

**Results:**

The study cohort had a mean age of 13.7 ± 2.6 months and an average follow-up of 58.4 ± 13.5 months. CR was successful in 50 hips (86.2%), while 8 hips (13.8%) failed. Compared to failed cases, successful reductions showed significantly lower aAFD (2.4 ± 0.88 mm vs. 5.12 ± 1.70 mm, *p* < 0.05) and higher CAHI (83.4 ± 3.5% vs. 68.7 ± 4.9%, *p* < 0.05). AVN was observed in 10 hips (17.2%). Both aAFD and CAHI demonstrated strong intra- and interobserver reliability. ROC curve analysis showed excellent predictive accuracy for CAHI (AUC = 0.990) and aAFD (AUC = 0.968), with optimal thresholds aligning closely with the proposed cutoffs. Univariate analysis identified higher preoperative IHDI grade (*p* = 0.022) and more severe AVN (*p* < 0.01) as significant predictors of CR failure.

**Conclusions:**

Closed reduction with spica casting remains an effective treatment for DDH in toddlers. Postoperative MRI evaluation using only aAFD and CAHI offers a reliable and clinically applicable method for assessing reduction quality. Larger aAFD and lower CAHI values indicate a higher risk of reduction failure, making these indices valuable for postoperative assessment and decision-making.

## Introduction

Developmental dysplasia of the hip (DDH) is a common congenital disease of the musculoskeletal system in newborns, with a range of severity from a stable hip with a mildly dysplastic acetabulum to complete hip dislocation (1). The incidence of hip dislocation at birth is between 1 in 1,000 and 5 in 1,000 and varies by region, ethnicity, etc., and the incidence of subluxation and dysplasia is approximately 10 in 1,000. However, when universal ultrasonographic screening is implemented, the reported incidence increases to between 25 in 1,000 and 50 in 1,000 (2–4). Early diagnosis and treatment of DDH are crucial for proper physiological maturation of the hip joint and prevention of early joint degeneration (5). In the 9–18 month age group, which is the toddler period, the traditional treatment for DDH is closed reduction (CR) and spica cast immobilization (6–8), although open reduction has been suggested by some scholars in recent years (9, 10). In our clinical setting, however, we routinely attempt closed reduction under general anesthesia for children aged 9–18 months, particularly in previously untreated cases. This approach remains common in many Asian and Eastern European centers, where access to early DDH screening is still developing and late-presenting cases are more frequent (8, 11, 12). Regardless of the treatment method chosen, timely and accurate assessment of the quality of CR is crucial for successful treatment and to avoid complications. At this stage, the hip's incomplete ossification and high cartilage content create challenges for traditional imaging methods. x-rays provide limited visualization of non-ossified structures (13), while CT scans expose patients to radiation and fail to depict cartilage adequately (5). Ultrasonography has been utilized for postreduction evaluation in DDH, there are significant variations in probe approaches and interpretation of results (14–16). Notably, hips are in a flexion and abduction position within spica casts, deviating from the standard position outlined in the extensively commanded Graf sonographic method. This divergence underscores the need for additional training and experience in utilizing ultrasonography for postreduction examinations. Furthermore, ultrasonography is limited in its ability to visualize bony structures and provide detailed imaging of soft tissues in the hip. Arthrography, though historically useful intraoperatively, cannot be repeated once a spica cast is applied and does not provide direct visualization of intra-articular structures (13, 17).

Magnetic resonance imaging (MRI) provides a noninvasive and highly detailed method for evaluating cartilaginous and soft tissue structures in DDH (18, 19). However, previous studies have employed a wide array of MRI parameters, sometimes more than ten, without establishing a universally accepted standard (19–23). Many of these indices are time-consuming and not directly reflective of reduction quality, limiting their practicality in routine clinical settings (24, 25). To address this gap, we propose a streamlined MRI-based approach that focuses on two key indices: axial acetabular femoral head distance (aAFD) (20, 21, 23) and coronal acetabular cartilage head index (CAHI) (22, 26). By validating the reliability and reproducibility of aAFD and CAHI, this study aims to introduce a concise, clinically applicable method for evaluating CR quality and identifying hips at risk of redislocation.

## Materials and methods

This study was approved by our institutional ethical committee (科2023-022-1). A retrospective analysis was performed on DDH patients aged 9–18 months who were treated with CR and Spica cast immobilization from June 2014 to June 2021. Demographic data and preoperative and postoperative x-ray and postoperative MRI data were recorded.

### Inclusion and exclusion criteria

**Figure 1:**
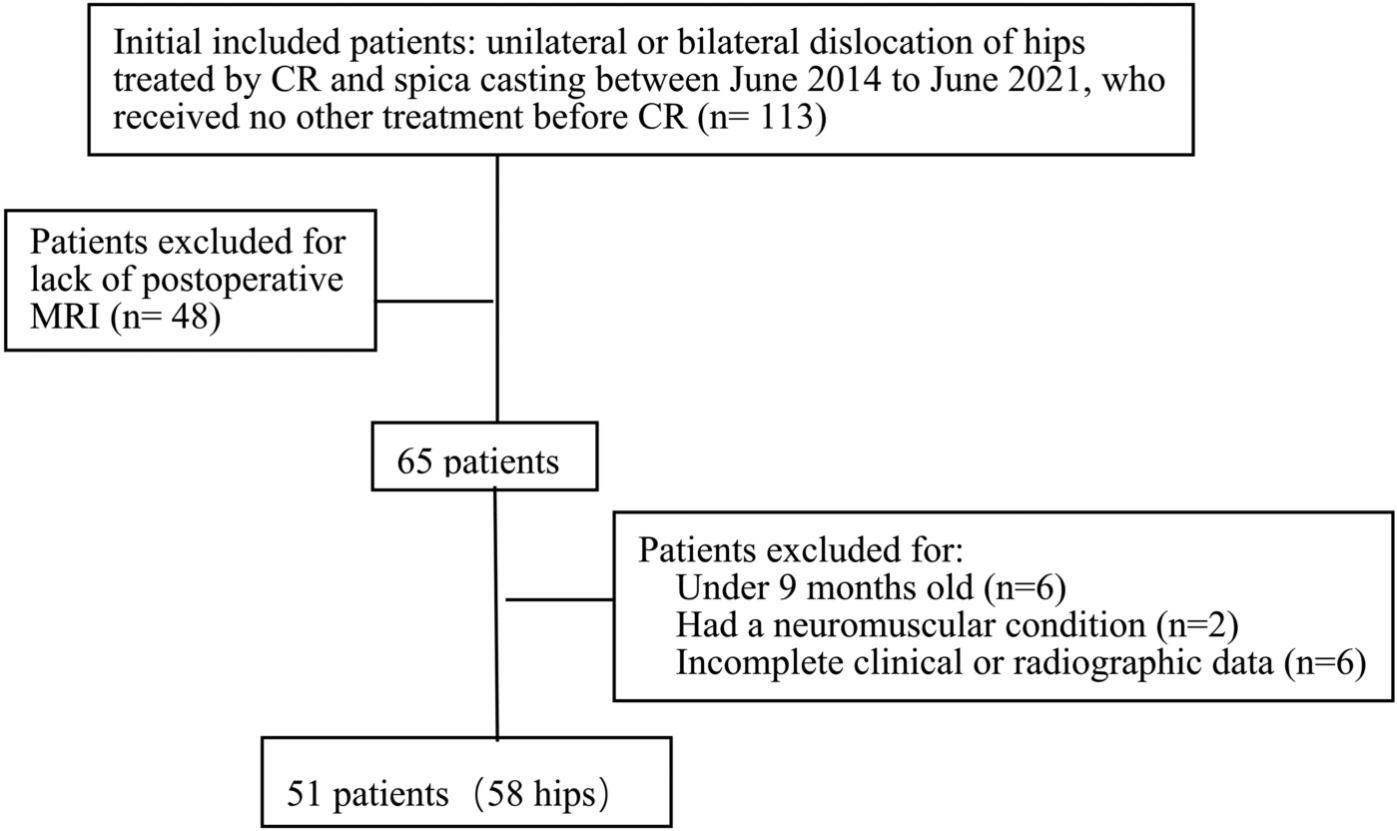
Patients flow chart.

The inclusion criteria were patients with unilateral or bilateral DDH aged 9–18 months treated by CR and spica casting, who received MRI assessment within 3 days after CR, and who received no other treatment before the operation. The exclusion criteria were children with inadequate clinical or radiographic data, other underlying diseases or syndromes associated with teratologic or secondary neuromuscular hip dysplasia, and a follow-up period of less than 2 years. Therefore, 51 patients (58 hips) with DDH were included in this study. The detailed data are presented in Figure 1. Contralateral healthy hips served as controls.

### Treatment

Closed hip reduction was conducted under general anesthesia with the patient in the supine position. To allow for a broader range of abduction and to enhance the stability of the reduction, the adductor muscle was released in cases exhibiting adductor tension before proceeding with closed reduction. In this series, 48 out of 58 hips underwent adductor muscle release. The patient was subsequently placed on a Spica frame, and hip reduction was achieved through flexion exceeding 90 degrees and gentle, gradual abduction while the greater trochanter was lifted. To establish a safe zone according to Ramsey's criteria (27), the hip was adducted to the point of redislocation, and the abduction angle of that position was recorded. The hip was then rereduced, and the abduction angle was recorded again. The disparity between these two angles defines the “safe zone,” with the accepted safe zone in our group set at 30 degrees or more. Upon confirmation of the range, a hip Spica cast was applied in the human position, with the hips flexed beyond 90 degrees and abducted at less than 70 degrees. If the CR fails to meet the specified criteria for the abduction angle and safe zone, which is indicative of unstable reduction, the procedure is discontinued. During the CR procedure, six out of 59 hips were discontinued because their failure to meet the specified criteria for abduction and safe zone angles. Additionally, two hips were identified as unreduced on the second-day postoperative MRI**.** These eight hips were subsequently treated with open reduction and consequently excluded from the statistical analysis.

Following the CR procedure, a post-Spica cast radiograph was acquired to confirm the initial positioning. Within three days postoperation, an MRI scan was conducted to assess the quality of the reduction. The Spica cast was typically worn for 12 weeks. Upon its removal, patients transitioned to a hip extension splint with 30–35 degrees of abduction for approximately 12 weeks, followed by a removable dynamic abduction brace for an additional 12 weeks of full-time use. Patients were then followed up at 3-month, 6-month, and 1-year intervals after the removal of the external fixator, with annual follow-up thereafter. At each follow-up, hip x-rays were performed to monitor the development of the hip joint, and both the joint's function and body images were documented.

### Overall examination indicators

All patients underwent hip x-ray examination before and after CR surgery, and the degree of DDH was classified according to the International Hip Dysplasia Institute (IHDI) classification (28). Postoperatively, the success of reduction was determined via hip MRI and x-ray, and the quality of reduction and prediction of late development of the hip joint were assessed according to the MRI indices described later. Patients who experienced redislocation during the follow-up period or those with residual dysplasia were categorized into the failure group. Follow-up was conducted for at least 24 months for all patients who did not experience redislocation or undergo a second surgery. AI measurements were conducted on hip plain films during the follow-up, and hips were appraised via the Severin classification system at the final assessment. The AVN condition of the femoral head was assessed according to the Kalamchi & MacEwen criteria (29). Residual dysplasia was defined as hips exhibiting an AI >2 standard deviations above the age-specific population-based mean value or a Severin classification grade of ≥3 at the last follow-up.

### MRI observation and measurements

All patients underwent bilateral hip MRI using a 3.0 T scanner (Siemens Magnetom Verio, Germany) within three days after closed reduction and spica cast application. Coronal and axial T1- and T2-weighted images were obtained. Chloral hydrate (50 mg/kg, per rectum) was used for sedation. MRI revealed various pathological features, including joint capsule thickening, inverted labrum, hypertrophic acetabular cartilage, and iliopsoas muscle atrophy.

Two key MRI parameters, aAFD and CAHI, were selected for quantitative assessment. Measurements were independently performed in a blinded manner by three trained pediatric orthopedic surgeons using standard PACS software with electronic calipers. To evaluate intraobserver reliability, one of the surgeons repeated the measurements at two separate time points. The average of the two reviewers' values was used for analysis.

**Figure 2:**
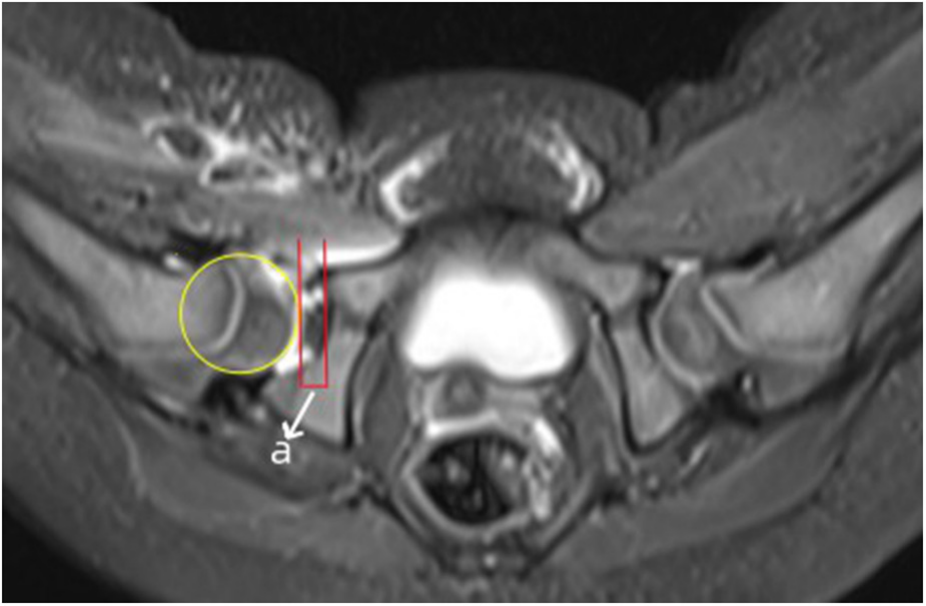
Measurement of aAFD (in mm) on an axial MR image. aAFD displaying the maximum diameter of the femoral head of a hip after CR and spica casting. The letter ‘a’ represents aAFD, which is the distance between the acetabular edge and the femoral head edge at the level of the femoral head center.

**Figure 3:**
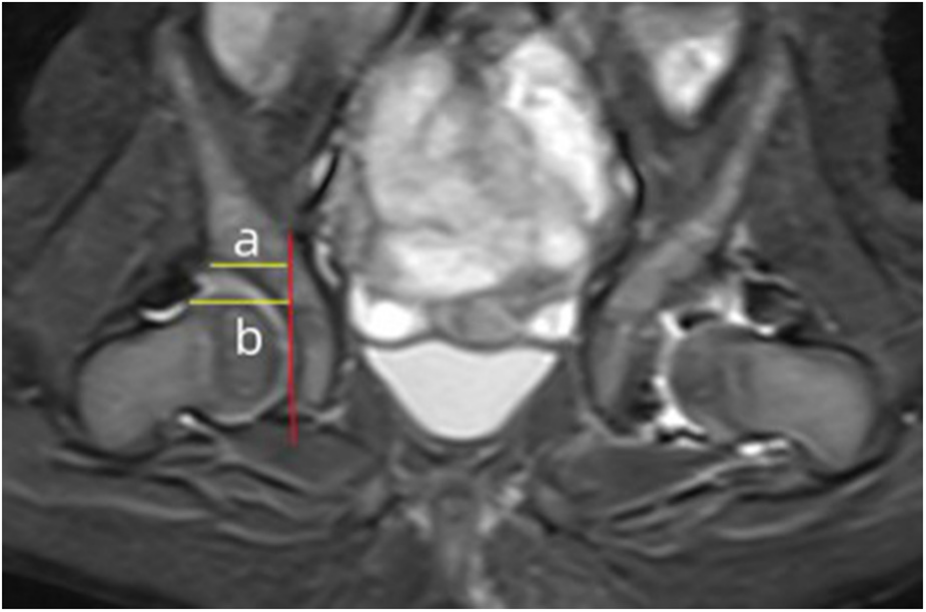
Evaluation of the CAHI. On the MRI coronal image showing the maximum diameter of the femoral head, a vertical line was drawn on the innermost edge of the femoral head cartilage. The vertical distance between the line and the outermost edge of the acetabular cartilage (a) and the distance between the line and the outermost edge of the femoral head cartilage (b) were measured. The percentage of these two distances is the CAHI (CAHI = a/b × 100).

The aAFD was measured on the axial slice showing the maximal femoral head diameter and was defined as the distance between the acetabular edge and the lateral margin of the femoral head at its center (Figure 2). The CAHI was measured on the coronal slice with the largest femoral head diameter (Figure 3). A vertical reference line was drawn along the innermost margin of the femoral head cartilage, and the CAHI was calculated as the ratio of the distance to the outermost edge of the acetabular cartilage (a) to that of the femoral head cartilage (b), using the formula CAHI = a/b × 100 (%).

CAHI was measured on coronal T2-weighted turbo spin-echo fat-suppressed images, while aAFD was measured on axial T2-weighted TIRM fat-suppressed sequences. Both sequences used a slice thickness of 4 mm and an inter-slice gap of 4.4 mm. To minimize selection bias, the slice with the most clearly defined femoral head and acetabular structures was chosen for each measurement. To further assess reproducibility, inter-slice variability was evaluated in 10 randomly selected hips: variation was within ±0.6 mm for aAFD and ±3.2% for CAHI, confirming acceptable measurement consistency.

### Statistical analysis

The data were analyzed via SPSS Statistics 25.0 (SPSS Inc., Chicago, IL). Descriptive statistics, including arithmetic mean values and standard deviations, were calculated. Data are given as the means ± standard deviations (SDs) and ranges, if not indicated otherwise. The nonparametric Mann‒Whitney U test was performed for metric, nonnormally distributed data. Student's t test was performed for normally distributed metric variables. Fisher's exact test was performed to analyze qualitative variables, including gender, affected side and preoperative IHDI grade. Any probability value of less than 0.05 was considered statistically significant. The inter- and intra-observer agreement was determined by calculating the intraclass correlation coefficient (ICC). The reliability was interpreted as poor (ICC = 0–0.2), fair (ICC = 0.3–0.4), moderate (ICC = 0.5–0.6), strong (ICC = 0.7–0.8) or almost perfect (ICC > 0.8).

## Results

### Patient data

This study included a total of 51 patients (58 hips) who underwent CR and spica cast immobilization. The participant demographics included three boys, 48 girls, 21 right hips, and 23 left hips, with seven cases involving bilateral hips. The average age of the cohort was 13.7 ± 2.6 months (range: 9–18 months). In terms of the preoperative IHDI grade, 6 hips were categorized as Grade II, 34 as Grade III, and 18 as Grade IV. The preoperative AI was 37.8° ± 5.3° (range: 31°–49°). CR achieved success in 50 hips (41 hips with Severin I and 9 hips with Severin II) reflecting an 86.2% success rate, whereas eight hips experienced failure. Among the failures, three hips exhibited redislocation (Severin VI), three hips demonstrated subluxation (Severin IV), and two hips were classified as Severin III at the final follow-up, resulting in a failure rate of 13.8%. The reliability coefficients for the Severin classification, concerning both intra-group and inter-group consistency, are 0.98 (95% CI: 0.98–0.99) and 0.96 (95% CI: 0.93–0.97), respectively, indicating strong agreement and suggesting high reliability of these variables. Among the 50 successfully treated hips, the minimum follow-up period was 49 months, with a mean follow-up duration of 58.37 ± 13.46 months (range 49–112 months). All the detailed data are presented in Table 1.

**Table 1:** Comparing of two groups about demographic data and results of closed reduction at final follow-up.

Parameter		SRH	FRH	*P*
Hips		50	8	
Age of CR (m)		13.5 ± 2.6 (9–18)	15.9 ± 1.1 (14–17)	0.52
Gender (male/female)		3/47	0/8	0.98
Lateral (left/right)		26/24	3/5	0.71
Prereduction IHDI grade (I/II/III/IV)		0/6/32/12	0/0/2/6	0.022
AI (°)	Prereduction	36.7 ± 4.8 (30–49)	37.8 ± 6.3 (31–49)	0.20
	Final follow-up	20.5 ± 4.6 (9–32)	36.3 ± 6.7 (46–28)	<0.05
AVN (I/II/III/IV)		7/1/0/0	0/0/2/0	<0.01
aAFD (mm)		2.4 ± 0.88 (1.02–4.12)	5.12 ± 1.70 (3.31–8.69)	<0.05
CAHI (%)		83.4 ± 3.5 (76.2–92.5)	68.7 ± 4.9 (60.1–76.0)	<0.05
Final Severin grade (I/II/III/IV–VI)		41/9/0/0	0/0/2/6	<0.01

aAFD, axial acetabular femoral head distance; CAHI, cartilaginous acetabular head index; SRH, successful reduced hips; FRH, failed reduced hips.

### AFD value after CR

A total of 102 hips (51 patients) were analyzed, with 58 dislocated hips and 44 contralateral stable hips. Postoperative MRI analyses were conducted for all patients, revealing that the aAFD in the successful CR group was 2.4 ± 0.88 mm (range: 1.02–4.12 mm), whereas it was 5.12 ± 1.70 mm (range: 3.31–8.69 mm) in the failure group. A significant difference between the two groups was observed (*P* < 0.05), but no significant difference was found between the contralateral stable hip and the CR successful hip (*P* = 0.083). The results are presented in Table 2. The reliability coefficients for these aAFD values concerning intrarater and interrater agreement are 0.78 and 0.73, respectively, indicating strong agreement regarding the reliability of these variables.

**Table 2:** Results of main MRI parameters for evaluating the quality of closed reduction of hips.

Parameter	SRH (*n* = 50)	FRH (*n* = 8)	CSH (*n* = 44)	*P* by t'test	
				SRH vs. CSH	SRH vs. FRH
aAFD (mm)	2.4 ± 0.88 (1.02–4.12)	5.12 ± 1.70 (3.31–8.69)	0.99 ± 0.20 (0.63–1.49)	0.083	<0.05
CAHI (%)	83.4 ± 3.5 (76.2–92.5)	68.7 ± 4.9 (60.1–76.0)	92.5 ± 2.6 (87.8–97.18)	0.544	<0.05

aAFD, axial acetabular femoral head distance; CAHI, cartilaginous acetabular head index; CSH, contralateral stable hips; SRH, successful reduced hips group; FRH, failed reduced hips group.

### CAHI values after CR

In the analysis of the CAHI on the coronal plane of postoperative MR images, the CAHI in the hips with successful CR was 83.4 ± 3.5% (76.2%–92.5%), whereas in the hips with failed CR, it was 68.7 ± 4.9% (60.1%–76.0%). A significant difference was noted between the two groups (*P* < 0.05). The CAHI was 9.8% greater in contralateral stable hips (CAHI = 92.5 ± 2.6, 87.8%–97.18%) than in successfully reduced hips. However, no statistically significant difference was detected between the contralateral stable hips and the reduced hips (*P* = 0.544) (Table 2). The reliability coefficients for CAHI values concerning intrarater and interrater agreement are reported as 0.79 and 0.75, respectively, indicating strong agreement regarding the reliability of these variables.

### X-ray results and complications

**Figure 4:**
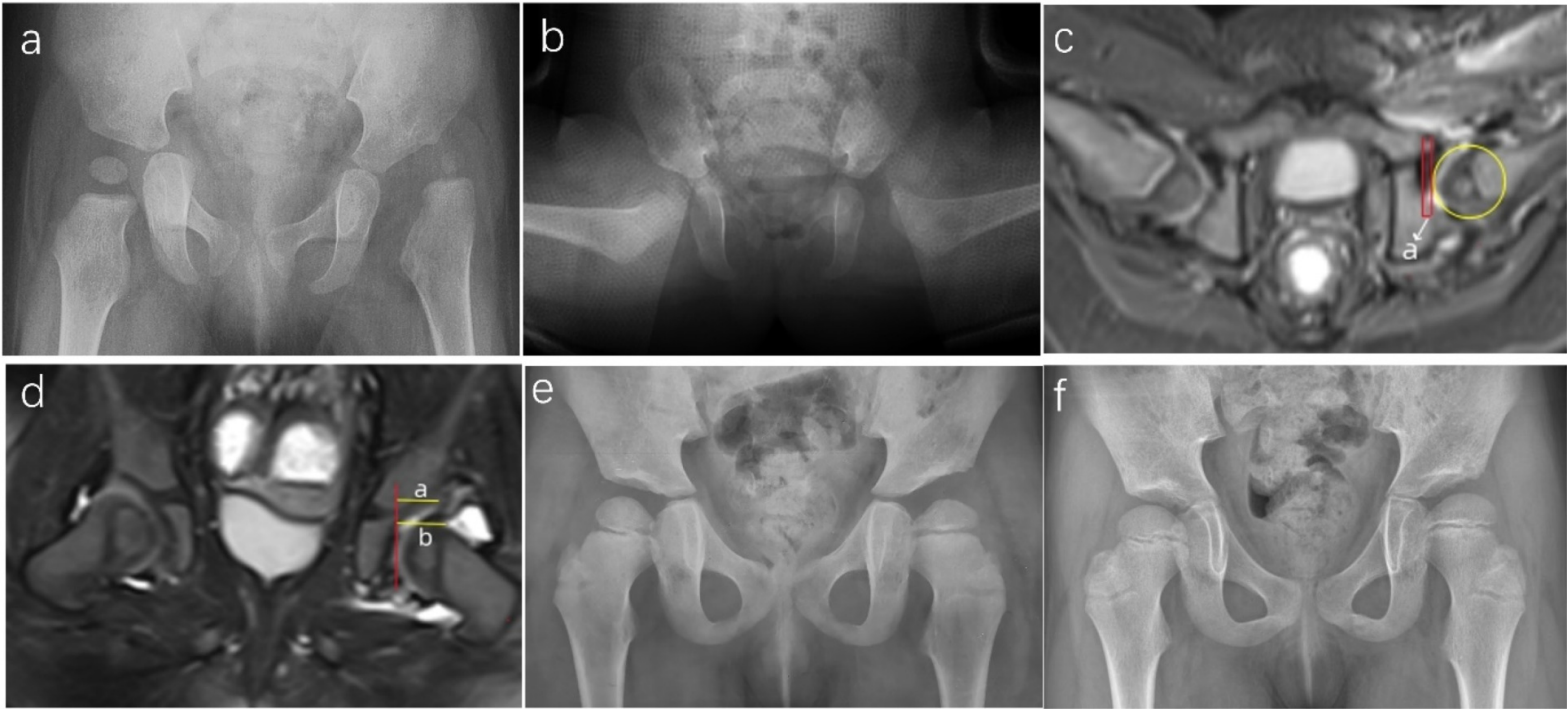
Representative case of successful closed reduction in a girl with left-sided DDH. **(a)** Preoperative radiograph of a 13-month-old girl showing left-sided DDH. **(b)** Immediate post-reduction radiograph following spica cast application. **(c)** Axial postoperative MRI demonstrating concentric reduction of the left hip, with an aAFD of 3.5 mm. **(d)** Coronal postoperative MRI showing a CAHI of 85% (calculated as a/b × 100). **(e)** Radiograph at 4 years of age (3 years post-reduction) showing normalization of the femoral head–acetabulum relationship. **(f)** Radiograph at 6 years and 7 months (5.5 years post-reduction), demonstrating a Severin Grade I outcome for the left hip.

**Figure 5:**
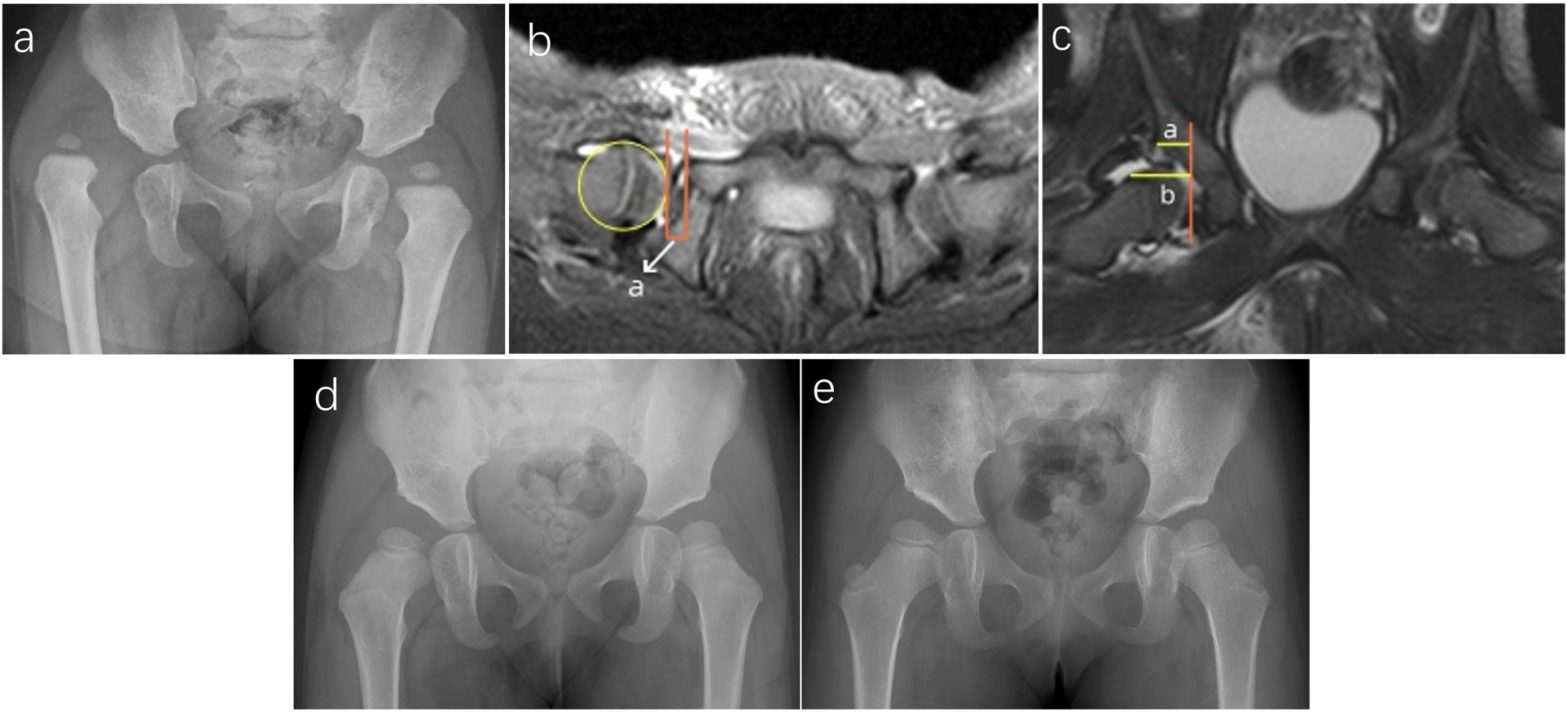
Representative case of failed closed reduction in a toddler with DDH, illustrating early MRI predictors of residual dysplasia. **(a)** Preoperative radiograph of a 15-month-old girl with right-sided DDH. **(b)** Postoperative axial MRI showing an elevated aAFD of 5.8 mm, indicating inadequate containment of the femoral head. **(c)** Postoperative coronal MRI demonstrating a CAHI of 56.7%, reflecting insufficient cartilaginous acetabular coverage. **(d)** Radiograph at 2.5 years of age showing a persistently shallow right acetabulum with an AI of 33°. **(e)** Radiograph at 4 years of age revealing continued acetabular dysplasia, with an AI of 31° and incomplete femoral head coverage, consistent with residual dysplasia and classified as Severin Grade III.

Among the 50 hips with successful CR, preoperative IHDI grading revealed 6 hips with grade II, 32 with grade III, and 12 with grade IV. The mean preoperative AI was 36.7° ± 4.8° (30°–49°), which decreased to 20.5° ± 4.6° (9°–32°) at the last follow-up. A representative case is illustrated in Figure 4. In the CR failure group, preoperative IHDI grading revealed 2 Grade III hips and 6 Grade IV hips. The mean preoperative AI was 37.8° ± 6.3° (31°–49°), and the last follow-up AI was 36.3° ± 6.7° (46°–28°), as detailed in Table 1. A representative case from the failure group is shown in Figure 5 to illustrate the imaging and clinical features of unsuccessful CR. No significant difference in the preoperative AI existed between the successful CR and failure groups (*P* = 0.20). During the follow-up period, AVN was observed in 10 out of 58 hips, which is 17.2% of the cases. According to the Kalamchi & MacEwen criteria, the AVN cases were categorized as follows: seven hips were Grade I, one hip was Grade II, and two hips were Grade III (Table 1). Among the 50 hips with successful closed reduction (CR), AVN was detected in eight hips—seven of which were Grade I, and one was Grade II. In contrast, among the eight hips that failed CR, there were two hips with Grade III AVN (Table 1). The AVN Grade results show coefficients of 0.98 (95% CI: 0.97–0.99) for intra-group consistency and 0.93 (95% CI: 0.89–0.96) for inter-group consistency, indicating strong agreement and confirming the high reliability of these variables. In the failure group, six hips underwent further surgical interventions, including pelvic osteotomy, proximal femoral osteotomy, and/or open reduction of the hip joint, and two hips in this group are currently under observation.

### Soft tissue observation via MRI

The conditions of reduction and the presence of soft tissue structures in coronary and axial plane MR images were analyzed. In the successful CR group, the soft tissues observed on MRI included an inverted labrum (four hips), thickening of the ligamentum teres (six hips), fibrofatty pulvinar tissue (two hips), and joint effusion (four hips). However, all these hips demonstrated positive outcomes. In the failure group, two hips presented fibrofatty pulvinar tissue, one presented joint effusion, and one presented an inverted labrum on MRI assessment of the hip joints.

### Risk factor analysis

Given the small sample size, a multivariate analysis to assess the interplay of risk factors for CR failure was not feasible. Consequently, we utilized chi-square tests and t-tests for a univariate statistical analysis of the data. This analysis indicated that a reduced CAHI and an increased aAFD were significantly associated with an elevated risk of CR failure (both *p* < 0.05) when compared to the stable contralateral hips, as detailed in Tables 1, 2. Furthermore, a higher preoperative IHDI grade (*P* = 0.022) and a more severe grade of AVN (*p* < 0.01) were identified as significant risk factors. In contrast, gender (*P* = 0.98), the affected side (*P* = 0.71), age (*P* = 0.52), and preoperative AI (*P* = 0.20) did not show a significant association with redislocation or residual deformities, as presented in Table 1. Abnormal soft tissues were observed in the hips on MRI in both the successful and failure groups. We could not obtain effective statistical results concerning whether soft tissues interfere with reduction because of the limited number of cases.

To further support the predictive value of aAFD and CAHI, ROC curve analysis was conducted. The area under the curve (AUC) was 0.990 for CAHI and 0.968 for aAFD, indicating excellent diagnostic accuracy. The optimal thresholds for predicting failure were determined as CAHI < 76.08% and aAFD > 3.935 mm, both closely aligned with the originally proposed values of 76% and 4 mm.

## Discussion

Early diagnosis and treatment of DDH are critical to avoid late complications such as abnormal gait, pain, and degenerative joint disease (1). However, in regions where screening for hip joint development is promoted late or slowly, there are still cases of late-onset DDH, and many cases are not identified until parents notice their child limping when practicing walking and seek medical attention (18, 19). This study focuses on a group of children aged 9–18 months with DDH who are at the walking stage. Unlike prestanding children whose hip joint pathological changes are relatively mild and can often be treated with Pavlik harnesses or abduction braces, and unlike older children with more severe pathological changes, such as joint capsule thickening, soft tissue contracture and fibrofatty pulvinar in the acetabulum, who may require open reduction and pelvic/femoral osteotomy, the treatment approach for this age group of DDH is unique. Generally, CR and Spica cast immobilization under general anesthesia are recommended for the walking age group (6, 7, 8), although there has been a trend toward more orthopedic surgeons performing open procedures recently (9, 10). In this study, we opted for CR and casting for this group. However, after hip reduction and cast placement, it is crucial to confirm the adequacy of reduction in a timely and accurate manner to determine whether to accept CR or switch to open reduction, which is necessary for successful treatment.

There are several options available to assess the adequacy of reduction in DDH, including intraoperative arthrography, x-ray, CT, MRI, and hip ultrasound, each with its own advantages and disadvantages. Pelvic radiographs were among the first medical images used to identify DDH, but their use in infancy is limited because of the absence of ossification or because the casts on the hips shield certain x-rays. CT scans are also not ideal, as they do not display cartilage well and involve more radioactive exposure (5, 13). Harcke et al. (14) examined hips via ultrasound after CR and spica casting and reported that ultrasound can reliably detect the femoral head position better than radiography can. However, it is limited by the acoustic window when it enters the spica cast, and the creation of a posterolateral window, while potentially beneficial for imaging, carries the risk of compromising the structural integrity of the cast. Recent reports have shown the use of transgluteal or transinguinal ultrasonography for post-reduction assessments, less common approaches in hip ultrasonography, that offer the advantage of preserving the integrity of the casting (15, 16). However, further research is needed before these methods can be widely adopted by practitioners. Arthrography, while traditionally valuable for intraoperative assessment of reduction adequacy, presents certain limitations. For instance, it is challenging to measure the acetabular femoral head space intraoperatively due to the magnification errors inherent in fluoroscopic images (21). Additionally, arthrography does not directly depict intra-articular structures or the depth of reduction, and it must be performed before Spica cast placement, limiting its repeatability once the cast is in place (13, 17). An additional concern is the risk of allergic reactions to the contrast medium used in arthrography, which, although rare, can be severe and even life-threatening (30). This highlights the importance of considering alternative methods for the assessment of hip reduction quality, especially when arthrography is contraindicated or cannot be performed.

MRI has several advantages over other imaging modalities, as it can reveal the immature femoral head, cartilaginous anlage of the acetabulum, and soft tissue and bone structures, enabling accurate determination of the center of the femoral head, regardless of the presence of the ossific nucleus (18, 19). Therefore, an increasing number of orthopedic surgeons are choosing MRI to confirm stable reduced retention and evaluate soft tissue interposition, although, there are several disadvantages associated with MRI, such as increased cost, increased time required to perform the examination, and the need for sedation. There is currently a lack of consensus on the acceptable limits of MRI indices for CR or which MRI indices may help determine the prognosis or risk factors for residual dysplasia. Several studies have used MRI to assess the quality of reduction and the prognosis of CR treatment for DDH, investigating over a dozen MRI parameters in total, with some studies evaluating as many as 5–7 indices in a single study (19–23). These indices encompass measurements of femoral head and acetabulum congruence and containment, as well as hip joint coronal and axial angles and indices, and the presence of soft tissue interposition in the relocated joint. However, investigating so many indices is clinically impractical and time-consuming, and some of them do not reflect the quality of hip reduction (24, 25). Our study simplifies MRI assessment by selecting only two parameters, the axial MRI index (aAFD) and the coronal MRI index (CAHI), to evaluate the quality of CR and assess its reliability and practicality. Several studies have employed either the CAHI or aAFD but have combined them with several other MRI indices to investigate the quality of CR (20, 22, 23, 26). Notably, the acceptable range of aAFD immediately after CR varies among different practitioners. Gans et al. reported that well-reduced cases had an acetabulum-to-femoral head distance of less than 2.8 mm on the axial plane. In contrast, Talathi et al. found an average distance of 3.5 ± 1.8 mm immediately following CR, which significantly decreased to 2.1 ± 1.1 mm by the third week post-surgery. In our study, in the successful group, the aAFD was under 4.12 mm, and 54% (27/50) of the hips had aAFD values greater than 2 mm. These findings suggest that generally successful CR procedures may lead to further improvement in the positioning of the femoral head over time, a phenomenon previously described as the “docking” phenomenon (23, 31).

Importantly, CAHI appears to be a more sensitive predictor than aAFD, as two failed cases in our cohort had aAFD values below the threshold but consistently low CAHI values (<76%). This finding supports the combined use of these indices for more accurate postoperative evaluation. We advocate for close monitoring or reconsideration of CR when CAHI falls below 76%, even if aAFD remains under 4 mm. To further validate the reliability of these thresholds, we conducted ROC curve analysis, which showed excellent predictive accuracy (AUC 0.990 for CAHI and 0.968 for aAFD). The optimal cutoff values identified (CAHI < 76.08%, aAFD > 3.935 mm) were nearly identical to our proposed thresholds, reinforcing their clinical applicability despite the small number of failure cases. Further research with larger, preferably multicenter cohorts is needed to refine these threshold values and validate their broader clinical applicability.

The incidence of AVN following CR and spica casting for toddler DDH remains a significant concern (32, 33). Prior studies, including that of Smith et al. (34), have suggested that excessive hip abduction-particularly angles exceeding 55–60°-may increase the risk of AVN. However, this association remains debated, with more recent literature, including MRI-based studies in children older than 6 months, reporting no clear correlation between abduction angles up to 70° and AVN risk when reductions are performed gently and within a safe range (8, 11, 31). In our cohort, closed reductions were performed without forceful abduction, and a minimum “safe zone” of ≥30° was ensured. Most hips were immobilized at <60° abduction, and only a few approached the 70° upper limit. Notably, the observed AVN rate of 17.2% did not appear to cluster at higher abduction angles and was comparable to rates reported in similar studies (12, 35, 36).

While the relationship between abduction angle and AVN is clinically relevant, a systematic statistical analysis of this factor was not performed in our study, as the primary aim was to validate MRI-derived parameters (CAHI and aAFD) as predictors of reduction quality. A rigorous AVN risk factor analysis would require not only abduction angle but also a multivariate approach adjusting for confounders such as age, sex, IHDI grade, and duration of immobilization—elements that fall outside the current study's scope. Nevertheless, we acknowledge this limitation and recognize the importance of a prospective study to more comprehensively explore the multifactorial contributors to AVN following CR, which we hope to initiate in the future.

In this series, we encountered eight cases of treatment failure. Our statistical analyses revealed an elevated risk of CR failure in hips with a decreased CAHI compared to the stable contralateral hip, as well as in cases with increased aAFD, more severe dysplasia (IHDI type IV), and advanced AVN (Grade III). Age, sex, and preoperative AI did not significantly influence the risk of failure, which aligns with findings from previous studies. Type I AVN appeared to be a transient developmental disturbance that resolved without long-term consequences. Due to our limited sample size, these risk factors were analyzed using univariate methods; thus, we were unable to explore the interrelationships among variables or identify which factors were independently predictive of treatment failure.

Furthermore, although the mean follow-up period of 58.4 months was sufficient to detect most clinically apparent AVN cases, we acknowledge the possibility of underestimating the true incidence, particularly in cases of Kalamchi–MacEwen type II, which may present later. Similarly, long-term outcomes such as Severin grade progression during adolescence and the potential for early-onset osteoarthritis remain beyond the current observational window. Our findings primarily reflect short- to mid-term hip morphology and joint congruency rather than definitive long-term joint function. While the early prognostic value of CAHI and aAFD appears promising, their predictive utility for durable joint health and skeletal maturity requires further validation in long-term longitudinal studies.

Additionally, postoperative MRI revealed abnormal intra-articular soft tissues, such as pulvinar remnants, inverted labrum, and joint effusion, in both successful (12 hips) and failed (3 hips) reduction groups. Although we sought to assess the contribution of these structures to reduction failure, limitations in sample size and image resolution precluded precise quantification of their impact. The clinical MRI protocol, while practical for routine assessment, lacked standardized imaging planes and sufficient spatial resolution for reliable volumetric evaluation of intra-articular morphology.

Taken together, these limitations, including retrospective design, modest sample size, lack of multivariate analysis, and the absence of long-term skeletal outcomes, constrain the strength of our conclusions. Nonetheless, our findings support the clinical utility of CAHI and aAFD as practical MRI-based indicators of reduction quality following CR in toddlers with DDH. Future prospective studies with larger, multicenter cohorts, high-resolution standardized MRI protocols, and extended follow-up are warranted to further validate these indices and refine treatment decision-making in this population.

## Conclusion

This study introduces a streamlined MRI-based method for assessing CR outcomes in toddlers with DDH. By focusing on two key indices -CAHI and aAFD - we provide a practical and reliable tool for postoperative evaluation. Successful reductions were associated with lower aAFD and higher CAHI values, highlighting the prognostic relevance of these parameters. Given their strong interrater reliability and predictive performance, CAHI and aAFD may serve as valuable adjuncts in routine postoperative assessment. Future prospective studies are needed to validate these findings across larger populations and to refine the threshold values for broader clinical application.

## Data Availability

The original contributions presented in the study are included in the article/Supplementary Material, further inquiries can be directed to the corresponding author.
